# Maternal Diet Supplemented with Methyl-Donors Protects against Atherosclerosis in F1 ApoE^−/−^ Mice

**DOI:** 10.1371/journal.pone.0056253

**Published:** 2013-02-21

**Authors:** Colin Delaney, Sanjay K. Garg, Chris Fernandes, Mark Hoeltzel, Robert H. Allen, Sally Stabler, Raymond Yung

**Affiliations:** 1 Department of Internal Medicine, University of Michigan Medical School, Ann Arbor, Michigan, United States of America; 2 Department of Pediatrics, Children’s Mercy Hospitals and Clinics, Kansas City, Missouri, United States of America; 3 University of Colorado School of Medicine, Aurora, Colorado, United States of America; University of Leicester, United Kingdom

## Abstract

Atherosclerosis is an inflammatory condition of the arterial wall mediated by cells of both innate and adaptive immunity. T lymphocytes play an important role in orchestrating the pathogenic immune response involved in the acceleration of atherosclerosis. Previously, we have shown that a prenatal methyl-donor supplementation diet (MS), when fed to dams during pregnancy and lactation, decreased the T cell-mediated pro-inflammatory cytokine and chemokine response in F1 mice. In the current study, we report feeding Apolipoprotein E (ApoE^−/−^) deficient dams with the MS diet during pregnancy reduces atherosclerotic plaques in F1 mice that were fed high fat diet (HFD) after weaning. F1 mice from dams on the MS diet exhibited increased global T cell DNA methylation. T-cell chemokines and their receptors (in particular CCR2, CCR5, and CXCR3) play important roles in the inflammatory cell recruitment to vascular lesions. MS diet significantly reduced Ccr2 mRNA and protein expression in CD3+ T cells but not in CD11b+ monocytes in MS F1 mice relative to controls. F1 litter size, HFD consumption, body weight, and body fat were similar between control and MS diet groups. Moreover, serum thiol metabolite levels were similar between the two groups. However, MS diet is associated with significantly higher serum HDL and lower LDL+VLDL levels in comparison to F1 mice from dams on the control diet. Inflammatory cytokines (IL-17, TNF-α, IL-6) were also lower in MS F1 mice serum and conditioned media from T-cell culture. Altogether, these data suggest that the MS diet ameliorates development of atherosclerosis by inhibiting the T-cell Ccr2 expression, reducing inflammatory cytokines production and increasing serum HDL:LDL ratio.

## Introduction

Atherosclerosis is a chronic disease that remains asymptomatic for decades [1]. Several pathogenic mechanisms have been proposed that contribute to the development of atherosclerosis. Both the pro-inflammatory cytokine (TNF-α, IFN-γ, IL-1, IL-6, IL-8, IL-17) and chemokine (CCR2, CCR5, MCP-1) systems are involved in the process [2], [3], [4], [5], [6], [7]. Abnormal metabolism such as hyperlipidemia and hyperhomocysteinemia is also associated with disease progression [8].

Epigenetic changes are heritable alterations in DNA that affect gene expression and function by mechanisms other than those from changes in DNA sequence. A number of epigenetic processes have been described including DNA methylation, chromatin remodeling, DNA acetylation, and by RNA splice variants. Alteration in DNA methylation is implicated in several human diseases ranging from cancer to heart disease and can be influenced by diet [9], [10], [11]. DNA methylation involves addition of a methyl group at the 5^th^-C position of cytosine residue in DNA which is catalyzed by DNA methyltransferases. S-adenosylmethionine, an intermediate product of methionine metabolism, acts as a methyl donor. Interestingly, pro-inflammatory cytokines, low folate intake, and hypercholesterolemia have been shown to alter the pattern of DNA methylation via epigenetic mechanism [9], [10].

Epigenetic patterns are primarily laid down *in utero*. Therefore, modifications of maternal diet in micronutrients that interact in the methionine cycle can lead to epigenetic differences in offspring [12]. A prime example of the effect of maternal methyl donor-based diet on F1 phenotype is provided by the viable yellow agouti mouse. In genetically identical mice, the agouti gene is differentially expressed due to epigenetic modifications established during early development that can persist through multiple generations, resulting in variations in coat color and susceptibility to cancer and obesity-related diseases [13], [14]. Waterland et al. recently suggested that rather than demonstrating inheritance of environmentally induced alteration in the Agouti epigenotype, maternal methionine supplementation may prevent the loss of existing epigenetic information during development [15].

Available evidence indicates that atherosclerosis is also related to aberrant DNA methylation [8], [11], [16]. In fact, epigenetic changes occur before the appearance of any histological detectable aortic lesions in 4 wk old ApoE^−/−^ mice [17]. We have previously demonstrated that prenatal diet supplementation with metabolites of the DNA methylation cycle increases global DNA methylation in T cells, decreases inflammatory cytokine release in mice and decreases the response to chemokine signaling *in vtiro* and *in vivo* without altering total CD3+ T cell number [18]. Although we showed that prenatal MS supplementation results in altered DNA methylation and T cell chemokine response [18], the effect on disease development is unclear. The purposes of this study is to determine whether methyl supplementation (MS) to dams could attenuate early atherosclerotic lesion in F1-ApoE^−/−^ offspring and explore possible mechanisms by which the MS diet affects development and progression of the disease.

## Materials and Methods

### Mice

Young adult (8–10 weeks) ApoE^−/−^ mice were purchased from The Jackson Laboratory (Bar Harbor, Maine). All diets were manufactured by Harlan Teklad (Madison, WI). Female mice were fed one of two synthetic diets, control or methyl-donor supplemented (MS) [18] (Table S1), for 3 wk to allow the mice to adjust, after which time the mice were mated. The MS diet was constructed based on the maternal diet that has previously been shown to alter DNA methylation in the offspring [18], and was reported to impact on DNA methylation through the methylation/methionine/folate cycle (Figure S1). Mated female mice remained on the diet throughout pregnancy and while lactating. At weaning male pups fed ad-libitum an adjusted calorie, 42% fat diet (HFD) until sacrificed at the appointed time points. All mice were maintained in a pathogen-free environment provided by the Unit for Laboratory Medicine at the University of Michigan. Procedures involving animals and their care were approved by the Committee on the Use and Care of Animals.

### Weight Gain, Chow Consumption, and Body Composition Analysis

F1 male mice were weighed at several time points to establish patterns of weight gain between control and MS diet groups. Consumption of chow per mouse per week was recorded. At 17, 28 and 34 wk of age, F1 mice underwent nuclear magnetic resonance (NMR)-based body composition analysis. Mice were placed in the measuring tube of a Minispec LF90 II (Bruker Optics, Germany) and their percentage fat, lean mass and fluid content determined.

### Splenocyte Separation and Purification

CD3+ T cells and CD11b+ monocytes were isolated by MACS microbeads technology (Miltenyi Biotec, Germany) from the spleen using negative and positive selection, respectively, according to the manufacturer’s instructions. Purity of the isolated cells was confirmed by flow cytometric analysis by staining with FITC-conjugated anti-CD3 or PE-conjugated CD11b, or FITC and PE-isotype IgG_2b,K_ Abs (BD Pharmingen, CA) and was consistently above 90%.

### Nucleic Acid Isolation

Total RNA was isolated from liver, splenic T cells and monocytes using RNeasy Mini (Qiagen, CA) according to the manufacturer’s instructions. Carryover DNA contaminants were removed using the RNase-Free DNase Set (Qiagen). Genomic DNA from livers and T cells was isolated using the GenElute Mammalian Genomic DNA Miniprep Kit (Sigma, MO). Livers were flash-frozen in liquid N_2_ and pulverized prior to genomic DNA preparation.

### Quantitative Real Time RT-PCR

q(RT)^2^-PCR was performed using QuantiTect SYBR Green RT-PCR Kit (Qiagen) according to manufacturer’s instructions. PCR reactions were carried out using a Rotor-Gene 6000 (Corbett Research, Australia). Data were analyzed using Rotor-Gene 6000 software, version 1.7. Expression levels of genes of interest were normalized to β-actin expression. Primer sequences used for qRT-PCR are shown in Table S2.

### Western Blot Analysis

Splenic T cells were isolated from 17, 28 and 34 wk old ApoE^−/−^ mice. Individual samples were pooled from 2–3 mice. Four million resting T cells were washed 2 times with cold PBS and lysed by suspending in lysis buffer as described previously [19]. 50 µg of total protein were loaded per well and separated on a reducing SDS-PAGE gel. CCR2 (Abcam, MA), levels in cell extracts was detected using the respective primary and secondary antibodies and detected using the chemiluminescent horseradish peroxidase system (Pierce, IL) as described previously [19]. The blot was then stripped using Restore Western Blot Stripping Buffer (Pierce, IL) and probed with actin (Santa Cruz, CA) and detected as described. The intensity of protein bands was quantified using the Image J software and normalized to the actin level in the same sample.

### Global DNA Methylation Analysis

5×10^5^ freshly isolated splenocytes were subjected to indirect fluorescent staining for 5-methylcytidine as described previously [18]. Briefly, splenocytes were stained with FITC-conjugated anti-CD3 and PerCP-Cy5.5-conjugated anti-CD11b antibodies or FITC and PerCP-Cy5.5 isotype Rat IgG_2b,K_, respectively (BD Biosciences, CA), then fixed in Cytofix/Cytoperm (BD) and permeabilized using PBS supplemented with 0.1% saponin, 1% FBS, and 0.1% sodium azide. Cells were then treated with RNase A to eliminate the potential for detection of 5-methylcytidine in tRNA. Cells were exposed to anti-5-methylcytidine (Acris Antibodies, Germany) in permeabilization buffer, washed, then incubated with PE-Anti-mouse I_g_G1 or PE-isotype rat-I_g_G1κ, (BD). Flow cytometry was performed immediately or within 24 hours of permeabilization using a FACScaliber machine (BD). Results were analyzed with FCS Express software (De Novo Software). T cell and liver DNA methylation was also quantified with the MethylFlash Methylated DNA Quantification Kit following manufacturer’s instructions (Epigentek).

### Aorta Plaque Quantification

At the time of death, mice were perfused first with PBS then with 10% Zinc (Fisher Scientific) Formalin. Aortas were excised, opened to expose the lumen, and stained with 0.2% Oil Red O dye (Sigma). Aortas were then pinned in dissecting plates, photographed and the ratio of atheroma surface area to total aorta surface area was quantified by a blinded observer using ImageJ software (http://rsbweb.nih.gov/ij/).

### Serum Metabolite Analysis

Blood was collected via orbital bleed at the time mice were sacrificed. Blood was allowed to coagulate for at least 30 minutes on ice, after which serum was isolated via centrifugation at 5000 RPM (2700 × g) for 15 minutes at 4°C. Serum was stored in aliquots in −70°C until use. Serum thiol metabolites such as homocysteine, cystathionine, cysteine, and methionine were assayed by capillary stable isotope dilution gas chromatography-mass spectrometry as previously described [20], [21]. Serum cholesterol level was measured by the HDL and LDL/VLDL Cholesterol Quantification Kit as per the manufacturer’s recommendation (Biovision, CA).

### Cytokine Measurement

Splenic CD3+ T cells were stimulated with 1 µg/mL ConA (Sigma) in the presence of 10 ng/mL IL-2 (R & D Systems, MN) and incubated at 37°C for 72 hr, at which time conditioned media were collected. Production of cytokines in the conditioned media and serum was measured using the Th1/Th2/Th17 Cytokine Kit (BD). Capture beads were detected with a FACSVantage flow cytometer (BD) and analysis was performed using FCAP Array Software Version 3.0 (BD). Flow cytometry and analysis was performed by the University of Michigan Flow Cytometry Core.

### Statistical Analysis

Mean and SEM were calculated using Microsoft Excel application software. Statistical significance was determined using one-tailed Student’s t test for single comparison. P values of <0.05 were considered significant.

## Results

### MS F1 Mice have Less Atherosclerotic Lesions in the Aorta than Control Mice

**Figure 1:**
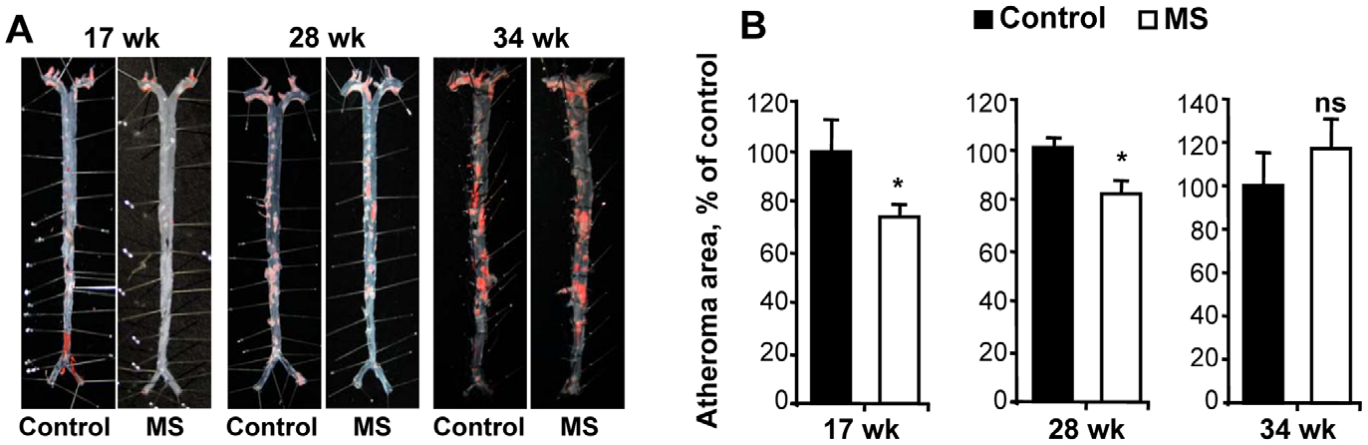
MS F1 mice have less atherosclerotic plaque in the aorta than control mice. (**A**) Representative images of control and MS F1 aortas at 17 wk, 28 wk and 34 wk of age. Oil Red O color indicates presence of atheroma. Panels **B** shows quantification of plaque surface area as a percentage of total aorta surface area in 17 wk, 28 wk and 34 wk F1 mice. Data presented is mean percent of control ± SEM. 17 wk: N = 5 control and 5 MS mice; 28 wk: N = 9 control and 7 MS mice; 34 wk: 7 control and 7 MS mice *p<0.05, ns = not significant.

We measured the surface area of atherosclerotic plaque relative to total surface area in formalin-perfused aortas. MS F1 mice had a statistically significant reduction in plaque formation in comparison to control F1 mice (Figure 1). MS F1 mice had 27% less plaque at 17 wk of age and 18% less plaque at 28 wk of age (Figure 1). However, by 34 wk of age, atheroma size in MS or control F1 mice were similar, suggesting that prenatal epigenetic-mediated protection can be diminished by chronic exposure to postnatal high fat diet.

### MS dams Gain Similar Weight and Give Birth to Similar Number of Pups When Compared to Control Dams

**Figure 2:**
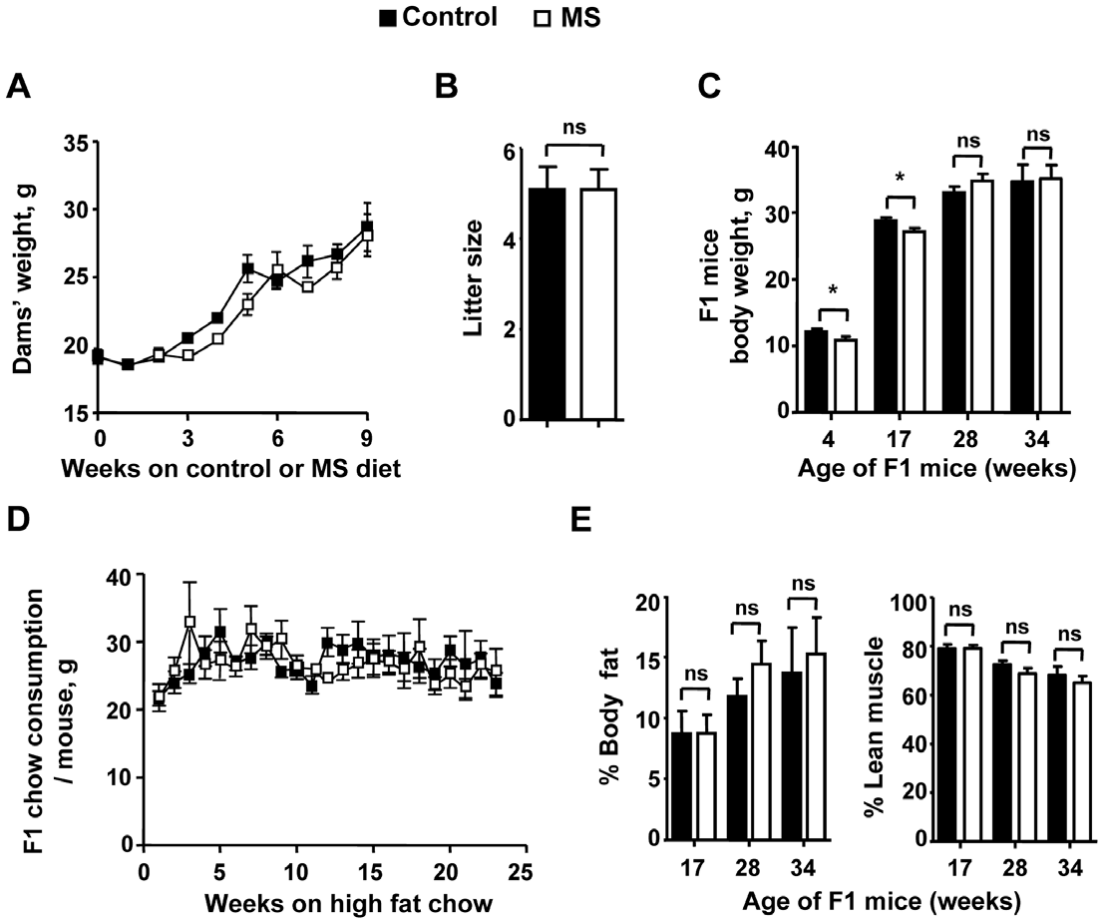
Weight, chow consumption, pup litter size and body composition of dams and F1 mice. A. Weight changes of dams over the weeks following mating. **B.** Pup litter size from dams on the control and MS diets. **C.** Body weight of control and MS F1 mice over time. Pups from control and MS fed dams were weaned at 4 wk of age onto a high fat (42%) diet (HFD). Weight of mice was measured at 4 wk, 17 wk, 28 wk, and 34 wk of age. **D.** Amount of HFD consumed by F1 mice per week over a 24 week period. **E.** Body fat percentage of control and MS F1 mice over time as measured by NMR. Data presented is mean ± SEM. A) N = 5 control and 5 supplement dams. B) N = 23 control litters and 22 MS litters. C) 4 wk: N = 14 control and 13 MS mice; 17 wk: N = 23 control and 21 MS mice; 28 wk: N = 27 control and 18 MS mice; 34 wk: N = 9 control and 9 MS mice. D) Total chow consumed per week per cage was divided by the number of mice per cage to determine average chow consumed per mouse. N = 11 control and 15 MS cages containing 1–5 mice per cage. E) 17 wk: N = 10 control and 9 MS mice; 28 wk: N = 21 control and 18 MS mice; 34 wk: N = 9 control and 9 MS mice. *p<0.05, ns = not significant.

We checked the weight gain of dams and F1 offspring as a possible mechanism for differential atherosclerotic plaque size between the two diets. Less atherosclerotic plaque in MS diet group is not attributed to differential body weight of dams between the two diet cohorts. ApoE^−/−^ dams fed MS diet gained similar amount of weight prior to and after mating (Figure 2A), consistent with our previous results in C57B/6 mice [18]. Dams gave birth to a similar number of pups per litter (Figure 2B). However, F1 MS mice are 11% and 5.7% smaller than control mice at 4 wk and 17 wk, respectively, but catch up in weight by 28 wk of age and maintained a similar weight at 34 wk (Figure 2C).

### MS F1 Mice Consume Similar Amount of High Fat Diet as Control Mice

Because MS F1 mice are smaller than control mice at weaning, we measured the amount of high fat food consumed by these mice relative to control animals to see if less feeding explained the lower plaque levels. MS F1 mice ate similar absolute amount of food relative to controls (Figure 2D). Therefore, differing feeding habits does not explain the decreased atherosclerosis in MS F1 mice. As an additional control, we checked the total body composition by NMR and found no difference in percent body fat and percent lean muscle mass (Figure 2E) between control and MS F1 mice at either 17 wk, 28 wk or 34 wk.

### MS F1 T cells are Hypermethylated Relative to Control Mice

**Figure 3:**
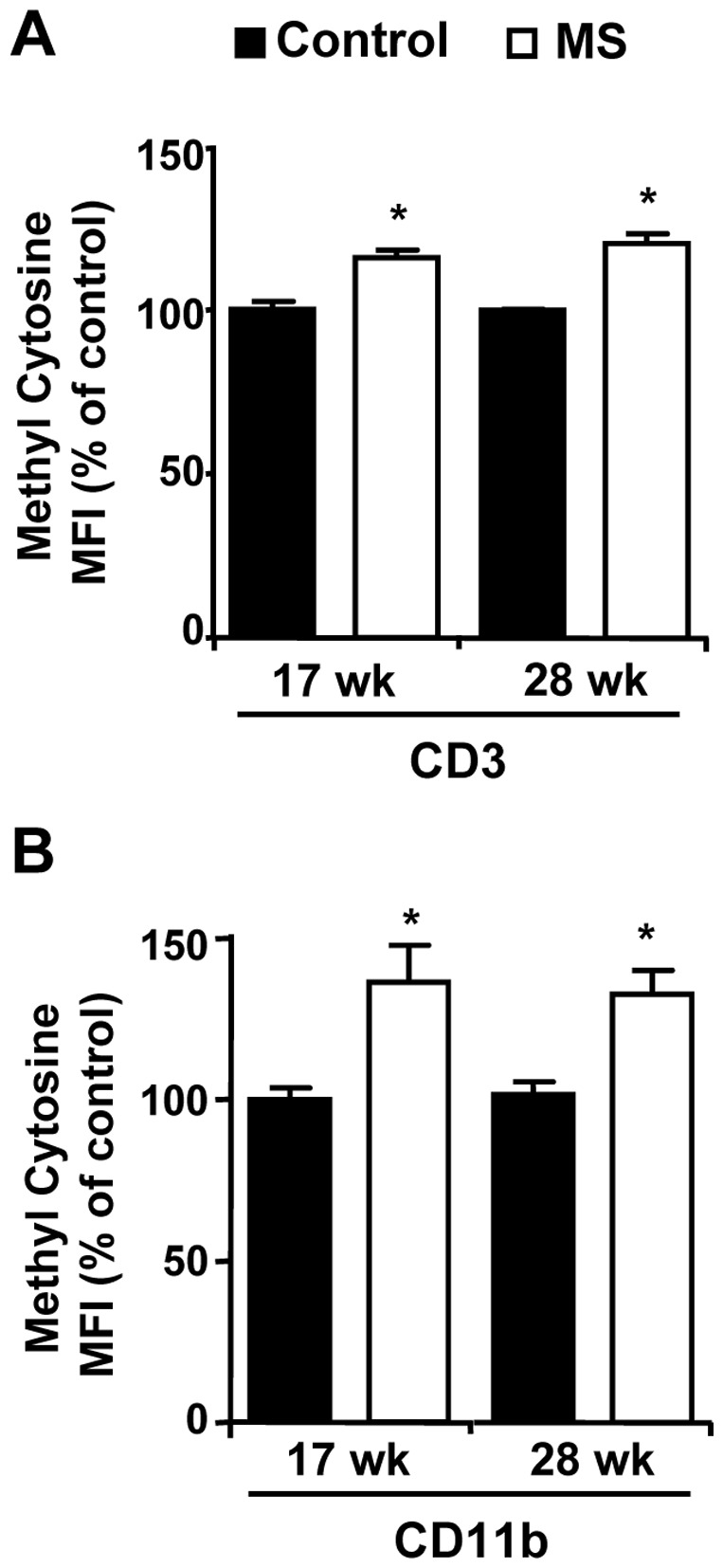
T cells and monocytes from MS F1 spleens are hypermethylated relative to control mice. CD3+ T cells (**A**) or CD11b+ cells (**B**) isolated from 17 wk or 28 wk old F1 ApoE^−/−^ mice were stained with anti-methylcytidine antibody and subjected to FACS analysis. Results shown are representative of 3 independent experiments. Data presented is mean ± SEM. 17 wk: N = 7 control and 5 MS mice; 28 wk: N = 9 control and 8 MS mice *p<0.05.

**Figure 4:**
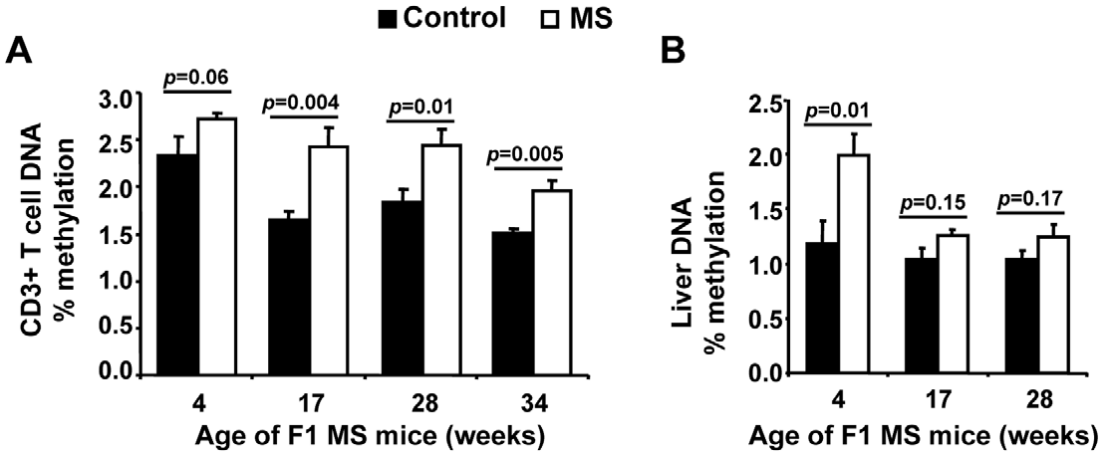
T cells and Liver from MS F1 mice are hypermethylated relative to control mice. T cell (**A**) and Liver (**B**) DNA were isolated from 4 wk, 17 wk, 28 wk or 34 wk old F1 ApoE^−/−^ mice and the level of methylation measured by ELISA. For each age group, the percent of methylated DNA (5-mC) in total DNA are shown. Results are mean ± SEM. (A) 4 wk: N = 5 control, 6 MS mice; 17 wk: 26 control, 23 MS mice; 28 wk: 28 control, 23 MS mice; 34 wk: 9 control, 9 MS mice. (B) 4 wk: N = 5 control, 6 MS mice; 17 wk and 28 wk: 10 control, 10 MS mice.

We quantified T-cell and monocytic DNA methylation with an unbiased, antibody-based flow cytometric method as shown in Figure 3. DNA from splenic CD3+ T cells from MS F1 mice had significantly higher methylation relative to controls at both 17 wk (16%) and 28 wk (20%) of age (Figure 3A). Similarly, DNA from CD11b+ cells was also found to be hypermethylated in MS F1 mice (Figure 3B). We confirmed MS diet-mediated hypermethylation of T cell DNA via ELISA (Figure 4A). Colorimetric based methylated DNA quantification also revealed a higher methylation of DNA in MS T cells in respect to control mice. DNA from MS T cells showed 16%, 45%, 32% and 29% increase in methylation at 4, 17, 28 and 34 wk of age respectively (Figure 4A). However, liver DNA showed a 68% increase in methylation at 4 wk of age in MS F1 mice and a trend toward hypermethylation at 17 wk that did not reach significance, while no difference was observed at 28 wk (Figure 4B). This suggests that methylation through MS diet may preferentially affect the immune system [22].

### MS F1 T cells Express Lower CCR2 than Control Mice

**Figure 5:**
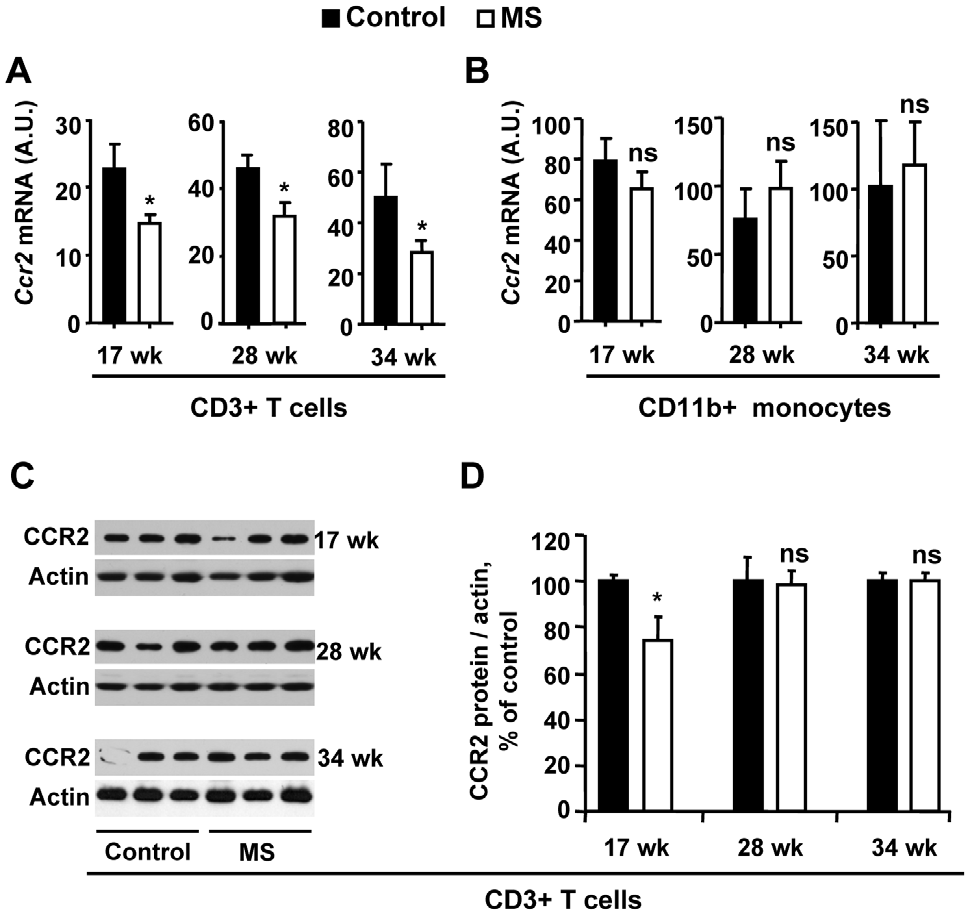
MS diet decreases CCR2 expression in F1 T cells. Splenic CD3+ T cells and CD11b+ cells from F1 ApoE^−/−^ mice were harvested at the age of 17 wk, 28 wk and 34 wk. mRNA was isolated and Ccr2 levels were measured by qRT-PCR in T cells (**A**) or monocytes (**B**). **C.** Protein from ApoE^−/−^ mice at the age of 17 wk, 28 wk, and 34 wk were used to check the level of CCR2 in CD3+ T cells. **D.** Quantitative analysis of panel C. Results are mean ± SEM. A, B, D) 17 wk: N = 26 control, 23 MS mice; 28 wk: N = 28 control, 23 MS mice. 34 wk: N = 9 control, 9 MS mice *p<0.05, ns = not significant.

Selected chemokine receptors have been implicated in the development and progression of atherosclerosis. We interrogated Ccr2, Ccr5, and Cxcr3 mRNA expression in T cells by qRT-PCR. T cell DNA global hypermethylation in MS F1 mice is accompanied by significantly lower Ccr2 RNA expression than controls at 17 wk, 28 wk, and 34 wk of age. The level of Ccr2 was 25%, 55%, and 52% lower at the age of 17 wk, 28 wk and 34 wk (Figure 5A), respectively. Ccr5 and Cxcr3 showed no difference between diet groups (Figure S2). In splenic monocytes, mRNA (Figure 5B) and protein (not shown) for these chemokines expression was not different between diet groups, with the exception of Ccr5 mRNA, which was upregulated in MS F1 monocytes at 28 wk of age. At the protein level, CCR2 expression was significantly (∼30%) lower in T cells from MS F1 mice in comparison to control at 17 wk but not at 28 wk or 34 wk (Figure 5C, D).

### MS F1 Mice have Higher Circulating HDL and Lower LDL/VLDL than Control Mice

**Figure 6:**
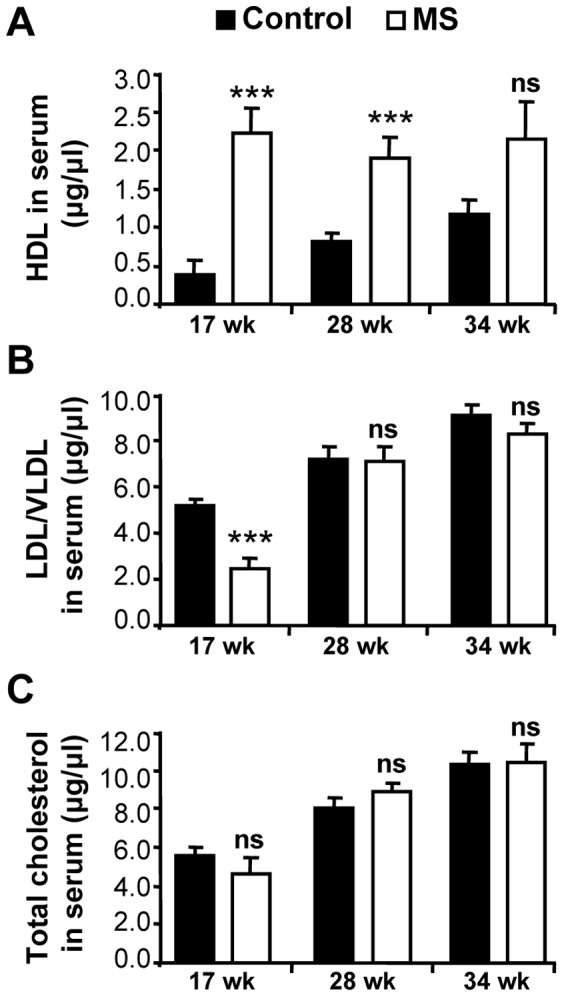
MS diet increase serum HDL and lowers LDL/VLDL in F1 mice. Serum was collected and cholesterol levels were measured fluorometrically as described in Methods. Results are mean ± SEM. 17 wk: N = 9 control and 9 MS mice; 28 wk: N = 11 control and 11 MS mice; 34 wk: N = 9 control and 9 MS mice. ***p<0.001, ns = not significant.

**Figure 7:**
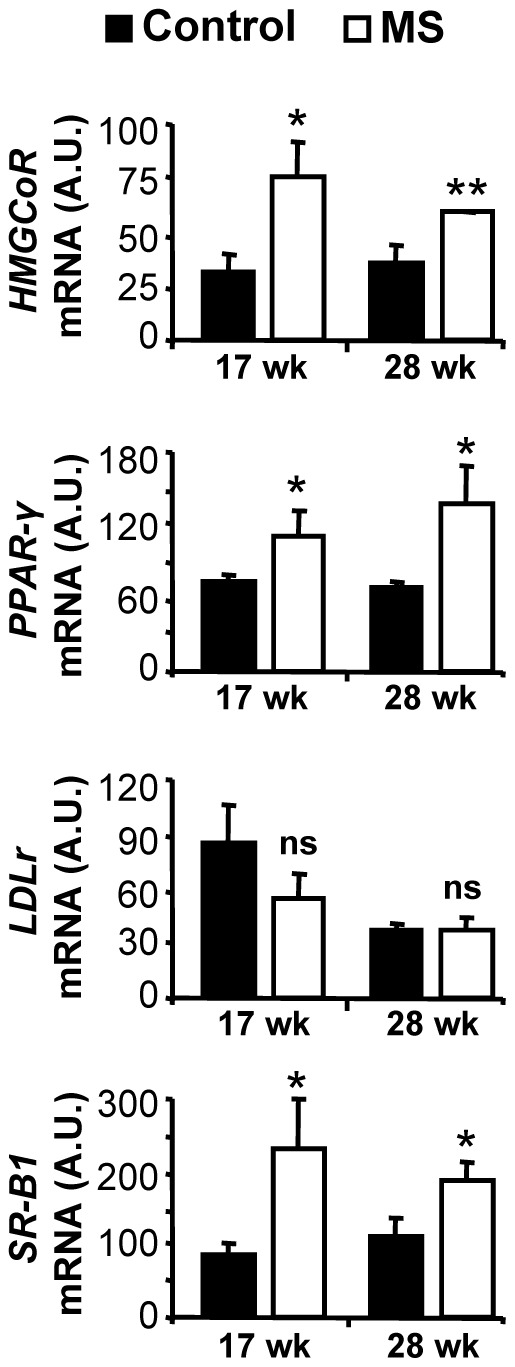
MS diet modulates the expression of hepatic genes involved in *de novo* synthesis, regulation, and transport of cholesterol. Liver mRNA from control and MS-F1 ApoE^−/−^ mice was harvested at the age of 17 wk, and 28 wk and levels of HMGCoR, PPAR-γ, LDLr, and SR-B1 were measured by qRT-PCR. Results are mean ± SEM. 17 wk: N = 7 control, 5 MS mice; 28 wk: N = 6 control, 5 MS mice. *p<0.05, **p<0.01, ns = not significant.

Hypercholesterolemia is seen as a key contributor to atherogenesis, so we interrogated cholesterol levels in the serum of the F1 mice. While total cholesterol levels were statistically similar between the F1 diet groups (Figure 6C), HDL was significantly higher in the MS F1 mice at both 17 wk and 28 wk of age (Figure 6A) but not at the age of 34 wk (Figure 6A). In contrast, LDL/VLDL levels were lower in MS F1 mice at 17 wk (Figure 6B), but were similar at 28 wk and 34 wk of age. We also checked the level of small thiol-containing metabolites in the serum such as homocysteine, methionine, cystathionine, and cysteine, they were not different between the two diet groups (Table 1). We checked the hepatic mRNA levels for proteins shown to be important in cholesterol uptake (LDLr, SR-B1), efflux (ABCA-1, CyP7a1), synthesis (HMGCoR), and regulation (PPAR-α, PPAR-γ, SREBP-1) via RT-qPCR [6], [23], [24]. While, no differences on the expression of ABCA-1, Cyp7a1, PPAR-α and SREBP-1 levels between control and MS-F1 mice were observed (data not shown), levels of SR-B1, HMGCoR and PPAR-γ expression were increased and LDLr expression was decreased in MS-F1 mice (Figure 7). MS-F1 liver had 165% and 65% higher SR-B1; 141%, and 56% higher HMGCoR; 55% and 95% higher PPAR-γ at 17 wk and 28 wk, respectively, relative to control-F1 mice. While level of LDLr receptor was decreased (35%) at 17 wk in MS-F1 mice, no difference at 28 wk was observed (Figure 7).

**Table 1:** Analysis of small thiol metabolites involved in methionine-homocysteine pathway in serums from 28-wk-old control or MS F1 mice.

**Metabolites**	**Control (n = 10)**	**Supplement (n = 10)**
Homocysteine, µmole/L	5.6±0.37	6.2±0.78 (p = 0.24)
Cystathionine, nmole/L	835±91	1029±91 (p = 0.12)
Cysteine, µmole/L	226±11	223±11 (p = 0.43)
Methionine, µmole/L	86.2±11.5	94.3±14.2 (p = 0.25)

N = 10 control and 10 MS mice.

### MS F1 Mice Produce Lower Level of Pro-inflammatory Cytokines than Control Mice

**Figure 8:**
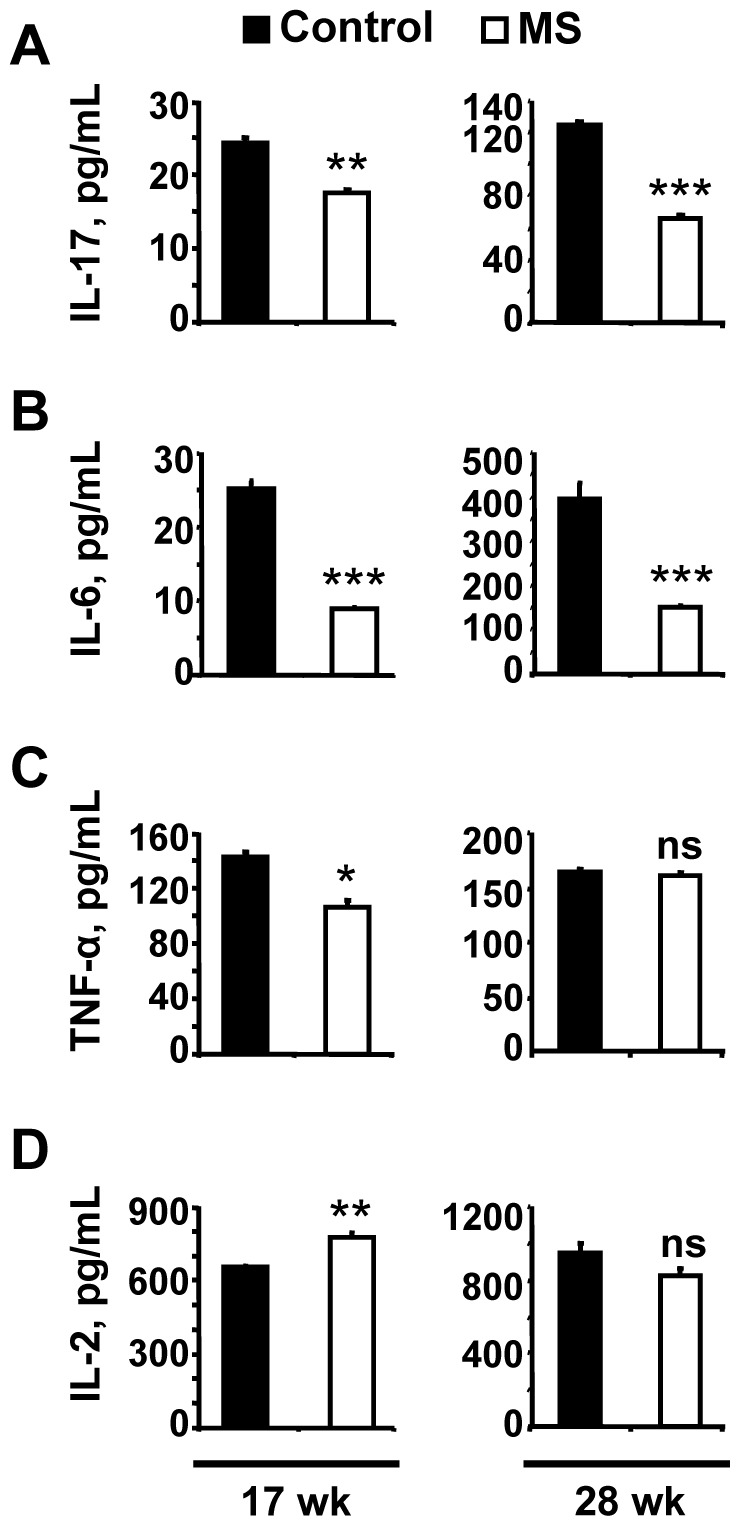
MS diet decreases production of pro-inflammatory cytokines in stimulated T cells. CD3+ T cells were stimulated with ConA in the presence of IL-2 for 72 h. The levels of IL-17, TNF-α, IL-6 and IL-2 in the conditioned media were measured as described in Methods. Results shows mean ± SEM and are representative of 2 independent experiments performed in triplicate. 17 wk: N = 5 control and 5 MS mice; 28 wk: N = 7 control and 7 MS mice. *p<0.05, **p<0.01. ***p<0.001.

**Figure 9:**
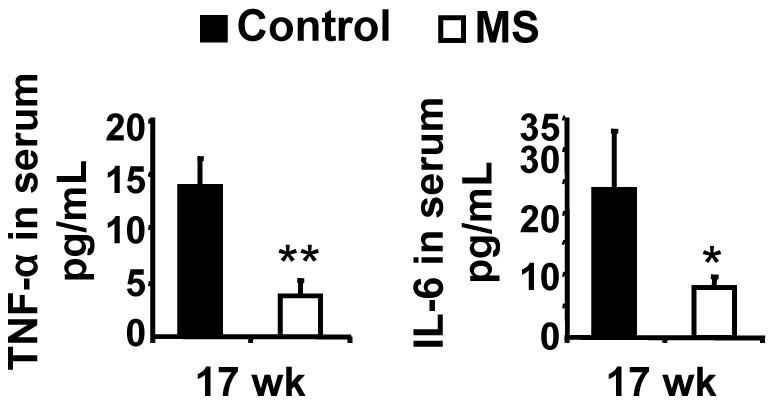
MS diet decreases production of pro-inflammatory cytokines in circulation. Serum was collected and the level of TNF-α and IL-6 was measured as described in Methods. Results are mean ± SEM. N = 4 control and 4 MS mice *p<0.05, **p<0.01.

Pro-inflammatory cytokines have been shown to accelerate atherosclerosis. We checked a panel of pro-inflammatory cytokines in serum as well as in CD4+ T-cell conditioned media from 17 wk and 28 wk old control and MS F1 mice. The T-cell conditioned media from MS F1 mice had significantly lower level of IL-17, TNF-α and IL-6 relative to control (Figure 8). IL-17 and IL-6 were 28% and 64% lower at 17 wk while 48% and 62% lower at 28 wk of age (Figure 8A, B). TNF-α showed 25% decrease at 17 wk, while no difference was observed at 28 wk of age (Figure 8C). IL-2 showed a modest 18% increase at 17 wk but showed a non-significant difference at 28 wk of age (Figure 8D). When we checked the level of TNF-α and IL-6 in the serum from 17 wk old ApoE^−/−^ control and MS F1 mice, a 73% and 66% decrease was observed in circulation, respectively (Figure 9).

## Discussion

Epigenetic regulation has been shown to be a powerful force in the mitigation or exacerbation of chronic diseases [24], [25], [26]. For example, dysregulation of DNA methylation has been implicated in cancer, neurodegenerative, cardiovascular and autoimmune diseases like lupus and rheumatoid arthiritis [11], [24], [25], [26]. However, the precise mechanisms linking epigenetic changes and disease are often poorly defined. Prenatal dietary micronutrient restriction has been used to induce hypomethylation in offspring, leading to over-expression of genes associated with stress, but this phenotype could be rescued with supplementation of the methylation pathway metabolite folic acid [12]. Prenatal diet supplementation with methyl donors can silence ectopic gene expression as seen in the viable yellow agouti mouse [15]. Our earlier work has demonstrated that a prenatal diet rich in methyl donors leads to an anti-inflammatory phenotype in wild-type C57Bl/6J mice [18]. Here we show that the same intervention protects against diet-induced atherosclerosis in the genetically predisposed ApoE^−/−^ mouse by decreasing systemic inflammation and increasing the HDL/LDL ratio.

MS F1 mice had a 18–27% reduction in aorta plaque relative to controls, which was not attributable to a difference in calorie or fat consumption. As had been previously observed in wild-type mice, this MS diet down-regulated the T cell expression of CCR2, the receptor for a key chemokine in atherogenesis, MCP-1, at both the RNA and protein level. Lower CCR2 expression may lead to less chemotaxis to the site of developing lesions, slowing the progression of disease. Previous studies have shown that CCR2^−/−^ApoE^−/−^ double knockout mice have less atherosclerotic plaques than single knockout mice [27]. Our diet-based intervention potentially offers a more feasible intervention in the prevention of atherosclerosis than gene-targeting methods.

We have previously reported that the MS diet induces hypermethylation in wild-type C57Bl/6J [18]. We see similar hypermethylation of DNA from splenic T cells and liver in 4 wk, 17 wk and 28 wk ApoE^−/−^ MS F1 mice. ApoE-deficient animals have been shown to have aberrant DNA methylation patterns prior to lesion formation [17]. Aging and autoimmunity are associated with T cell DNA hypomethylation [28], suggesting higher DNA methylation levels in T cells may be a ‘youthful’ or ‘healthy’ phenotype. Such an assertion matches our observations, as we find F1 ApoE^−/−^ mice with higher T cell global methylation have less plaque than controls. One interesting question yet to be addressed is whether the observed methylation levels in MS F1 mice is simply due to a higher starting pool of methyl-cytosine or if MS F1 mice are more successful maintaining methylation patterns than their control counterparts.

We observed a surprising difference in the retention of methylation patterns with age in liver and immune cells (Figure 4). Similar to our previous study in which we reported that T cell’s DNA from C57BL/6 MS F1 mice are hypermethylated at 4 wk of age relative to control mice [18], our current study also demonstrates that MS-mediated hypermethylation of DNA from T cells is maintained in 17, 28 and 34 wk old F1 mice. Interestingly, we found a similar hypermethylation in liver DNA at 4 wk, but this difference was not maintained at later ages. Comparable to our observations, Ono et al also demonstrated tissue-specific DNA methylation patterning in spleen and liver cells in aging mice [29]. This differential epigenetic plasticity may be due to differences in maturation between the two tissues. Cells of the immune system are generated by hematopoetic stem cells (hSCs) which develop during gestation and self-renew as they give rise to myeloid and lymphoid cell lineages [30]. Maintenance of epigenetic programming is key to retaining multipotency, since alterations of DNA methylation are known to trigger differentiation toward immune cell precursors [22]. In contrast, postnatal liver growth is primarily accomplished via somatic cell division, even though a small pool of hepatocyte precursors may play a role in repairing damage due to liver disease [31]. Indeed, liver regeneration proceeds via compensatory hyperplasia rather than through immature hepatocyte differentiation [32]. It follows that maintenance of epigenetic marks in somatic cell division of mature hepatocytes may be less critical than in the immune cell compartment, in which differentiation of hSCs is coordinated with alteration of DNA methylation.

Maternal weight gain during pregnancy has been associated with risk of heart disease in the offspring [33], [34]. Our data showed that chow consumption and maternal weight during pregnancy were similar in dams fed the control or MS diet. This suggests that the protective effect of the MS diet in F1 generation is independent of maternal macronutrients consumption and weight gain. Childhood obesity also increases future risk of heart disease [35], [36]. Furthermore, the older the children are, the greater the likelihood for later heart risk. For example, a Danish study reported that a boy who was heavier than his peers at age 7 had a 5% increased risk for late life cardiovascular disease, but a boy who was heavier than his peers at age 13 had a 17% greater risk [36]. Interestingly, the weight of the F1 offspring from dams on the MS diet was lower than the control cohort from 4 wk of age (equivalent to 2 years old humans) until they were 28 wk old (equivalent to 14 years old humans).

High cholesterol concentration and the nature of its esterification have been linked to risk of developing heart disease [6]. While high HDL levels are seen as protective, high LDL and VLDL concentrations are pro-atherogenic. Unexpectedly, our MS diet shifts the ratio of HDL:LDL towards more HDL in MS F1 mice while total cholesterol remains unchanged at 17 wk and 28 wk of age. This trend was also observed at 34 wk but did not reach significance (Figure 6C). LDL levels in MS F1 mice were initially low relative to controls at 17 wk of age, but there was no difference at 28 wk of age. The increase of ‘bad’ cholesterol with prolonged exposure to a high fat diet may be indicative of negative environmental factors eventually overwhelming *in utero* established epigenetic protections. Cholesterol esterification is controlled by the liver. We found MS F1 mice had hypermethylated liver DNA early in life. It is yet to be determined whether this difference in global methylation leads to altering expression of genes important in cholesterol metabolism, resulting in a protective phenotype. Among other hepatic genes known to be involved in cholesterol metabolism, PPAR-γ is a methylation sensitive gene. We observed an increase in the expression of PPAR-γ and SR-B1 mRNA in MS-F1 liver relative to control mice. PPAR-γ and SR-B1, reportedly a transcriptional regulator and HDL receptor, respectively, are also shown to protect against early-onset atherosclerosis [6], [23], [37]. Anti-atherogenic effect of PPAR-γ could be mediated by modulation of expression and secretion of paraoxonases, which is shown to decrease atherosclerotic lesions and inflammation [37]. Thiol serum metabolites that are involved in the homocysteine/methionine metabolism were not different between diet groups. Homocysteine levels were not elevated in either diet group compared to normal physiological level, suggesting that homocysteine is not playing a key role in this model of atherosclerosis, although it is possible they were exposed to higher levels of homocysteine *in utero* and prior to weaning. Interestingly, ApoE^−/−^ mice have a moderate decrease in plasma homocysteine and glucose levels yet develop severe atherosclerotic plaque [38]. Hyperhomocysteinemia has been reported to promote inflammatory monocyte generation and atherosclerosis acceleration [39]. The lack of change in homocysteine levels may explain the lack of difference in CD11b+ chemokine receptors expression between the two diet regimens. Prenatal MS diet is less effective at reducing plaque lesions, inflammation and LDL level the longer the mice are on the high fat diet, stressing the importance of healthy lifestyle in the maintenance of a beneficial epigenetic signature. This is consistent with the observation that established epigenetic markers in T cells (such as DNA methylation, histone acetylation) in monozygotic twins were found to correlate strongly in young twins (age 3 years) but these associations weakened with age and, presumably, lifestyle of the twins [40]. However, methyl donor supplementation has been shown to adversely affect health, notably in airway inflammation and inflammatory bowl disease (IBD) [41], [42]. Hollingsworth et al found that prenatal methyl donor diet exacerbated ovalbumin-induced airway inflammation in F1 mice. Also, Schaible et al showed that maternal methyl-donor supplementation led to increased offspring colitis susceptibility in mice. Surprisingly, Hollingsworth et al found no increase in global methylation levels [41], while Schaible et al analysed the methylation status of genes reported to be associated with IBD such as ptpn22 and Cpn2 and showed decreased methylation and increased expression with a significant inverse correlation in the F1 mice from methyl-donor supplemented dams [42]. Interestingly, both of these reports have different micronutrients supplemented at different levels. Our data show higher global methylation and suggest a beneficial relationship between methyl-supplemented prenatal diet and epigenetics in the development of atherosclerosis that can be lost by chronic exposure to poor diet.

There is an increasing interest in the role that cytokines play in controlling inflammation in murine models of atherogenesis [2]. Cytokines that have been shown to promote atheroma formation include TNF-α) [3], IFN-γ) [4], IL-1 [5], IL-6 [6], IL-17 [7] and IL-12 [43]. In contrast, IL-10 [44] is considered anti-atherogenic. For example, IL-6 over-expression in atheroma has been reported [6]. IL-6 can alter the pattern of protein biosynthesis in the liver, augmenting the synthesis of C-reactive protein, a marker of inflammation measurable in peripheral blood [45]. TNF-α plays a pleiotropic effect in the pathogenesis of several metabolic and inflammatory disorders, which are also risk factors for cardiovascular diseases. Atherosclerotic wall-thickening and lesion progression was lower in TNF^−/−/^ApoE^−/−^ double knock out mice [46]. Importance of diet and role of TNF-α in atherosclerosis progression was further substantiated by a study in which the authors showed that ApoE^−/−/^TNF-α^−/−^ double knockout mice had a 50% reduction in lesion size relative to ApoE^−/−^ single knockouts when put on ‘Western-style’ high-fat diet [3]. Similarly, IL-17 producing T cells have been identified in the human carotid artery plaques. Neutralization of IL-17A not only reduced the atherosclerotic plaques but also down regulated IL-6, TNF-α and CCL5 [7] that suggests that regulation of these cytokines may be interconnected in atherosclerosis. Pro-inflammatory cytokines may also alter the pattern of DNA methylation in cells. PPAR-γ controls the expression of paraoxonase that are shown to decrease inflammatory factors involved in the development of atherosclerosis (such as IL-1, IL-6 and TNF-α) [37], [47]. Induced up-regulation of PPAR-γ could be another mechanism by which the MS diet exerts a beneficial effect in atherosclerosis. We report that MS diet not only increased global DNA methylation but simultaneously decreased the LDL level and pro-inflammatory cytokines in T cell conditioned media (TNF-α, IL-6 and IL-17) and in serum (TNF-α and IL-6).

Our data show that a prenatal diet rich in methyl donors significantly retarded the pathogenesis of atherosclerosis in F1 mice through complementary mechanisms, by increasing DNA methylation of T cells, down-regulating the key chemokine receptor CCR2 and shifting the HDL:LDL serum cholesterol level. This protective effect is independent of homocysteine levels and lessens over time and eventually is overcome with chronic exposure of F1 mice to high fat diet. Our study demonstrates the outsized impact prenatal nutrition can have in modulating susceptibility to later-life chronic disease as well as limitations of such interventions when faced with persistent environmental insults.

## Supporting Information

Figure S1 **The relationship between the DNA methylation, methionine and folate cycles.** Boxes denote the ingredients that were supplemented to the control diet.
(TIF)

Figure S2 **Effect of MS diet on CCR5 and CXCR3 expression in F1 T cells and monocytes.** Splenic CD3+ T cells and CD11b+ monocytic cells from F1 ApoE−/− mice were harvested at the age of 17 wk and 28 wk. mRNA was isolated and Ccr5, Cxcr3 levels were measured by qRT-PCR in T cells (A) or monocytes (B). Results are mean ± SEM. A, B) 17 wk: N = 20 Control and 17 MS mice; 28 wk N = 28 control and 23 MS mice. *p<0.05, ns = not significant
(TIF)

Table S1 **Diet composition.**
(PDF)

Table S2 **Primer sequenced used for RT-PCR.**
(TIF)
