# Supplementary figures and images for: Maternal Diet Supplemented with Methyl-Donors Protects against Atherosclerosis in F1 ApoE−/− Mice
Source: PLoS One. 2013 Feb 21;8(2):e56253. doi: 10.1371/journal.pone.0056253 (PMC3578836; doi:10.1371/journal.pone.0056253)

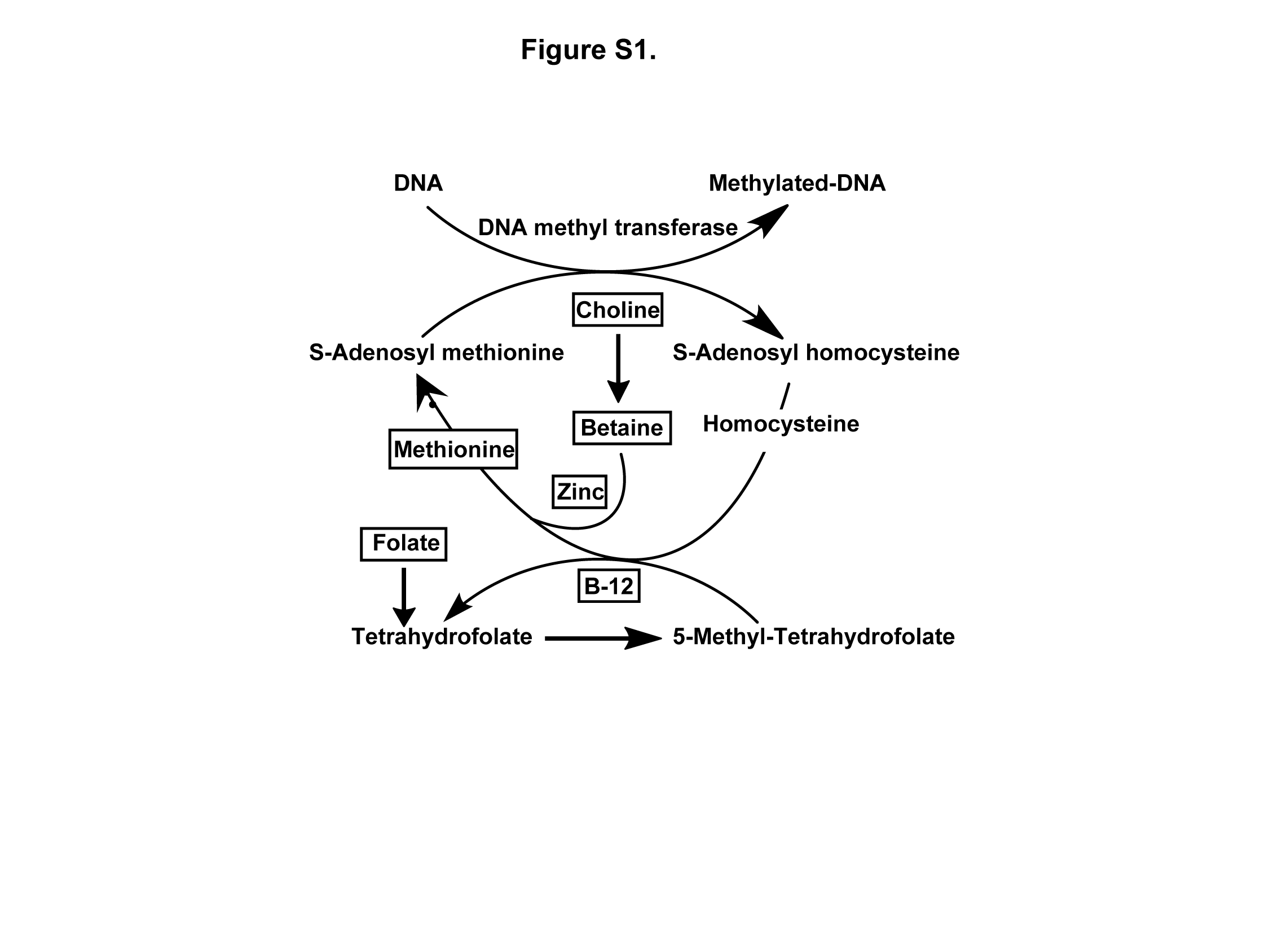

Supplement: Figure S1 — The relationship between the DNA methylation, methionine and folate cycles. Boxes denote the ingredients that were supplemented to the control diet. (TIF) [file pone.0056253.s001.tif]

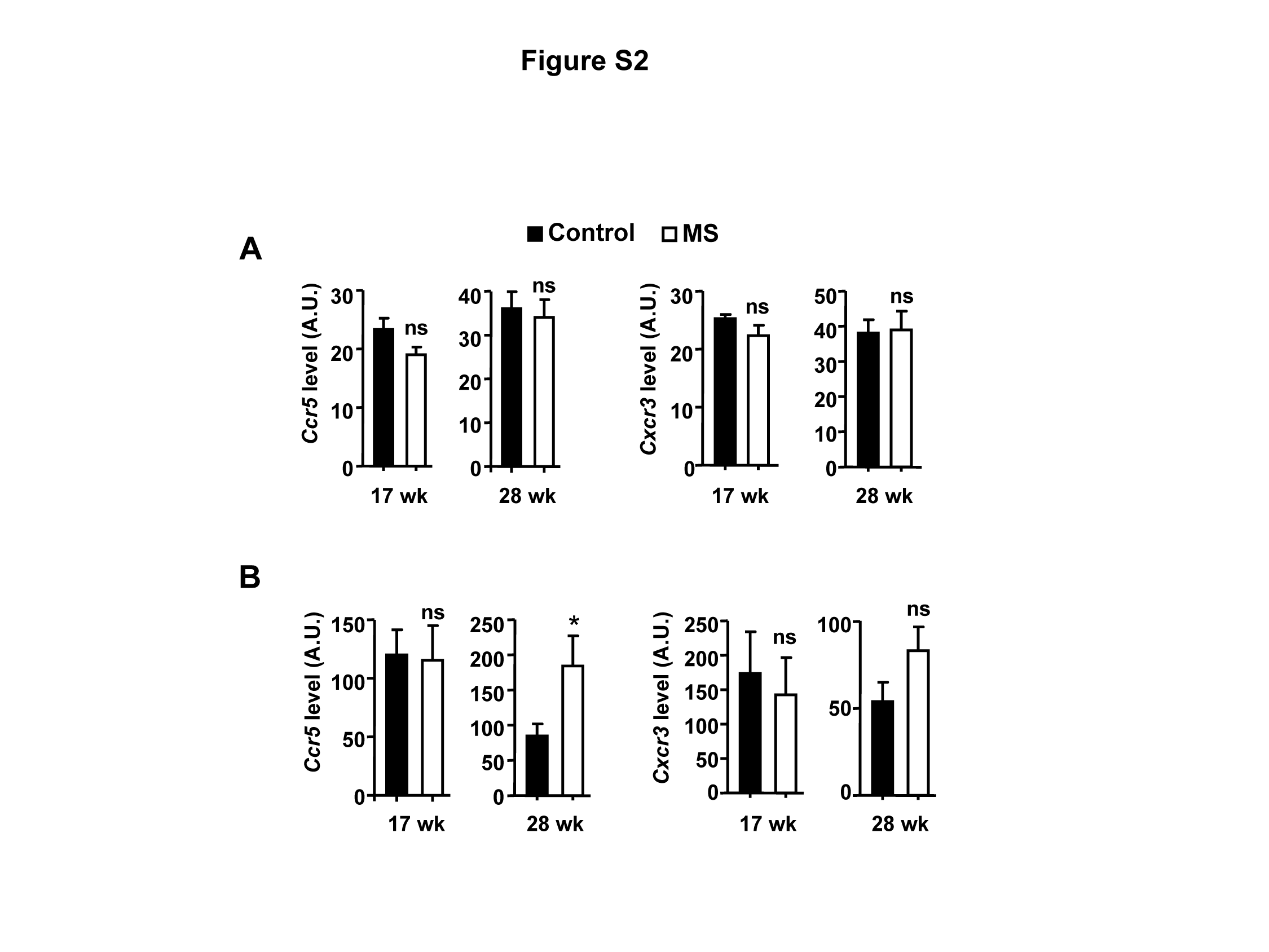

Supplement: Figure S2 — Effect of MS diet on CCR5 and CXCR3 expression in F1 T cells and monocytes. Splenic CD3+ T cells and CD11b+ monocytic cells from F1 ApoE−/− mice were harvested at the age of 17 wk and 28 wk. mRNA was isolated and Ccr5, Cxcr3 levels were measured by qRT-PCR in T cells (A) or monocytes (B). Results are mean ± SEM. A, B) 17 wk: N = 20 Control and 17 MS mice; 28 wk N = 28 control and 23 MS mice. *p<0.05, ns = not significant (TIF) [file pone.0056253.s002.tif]

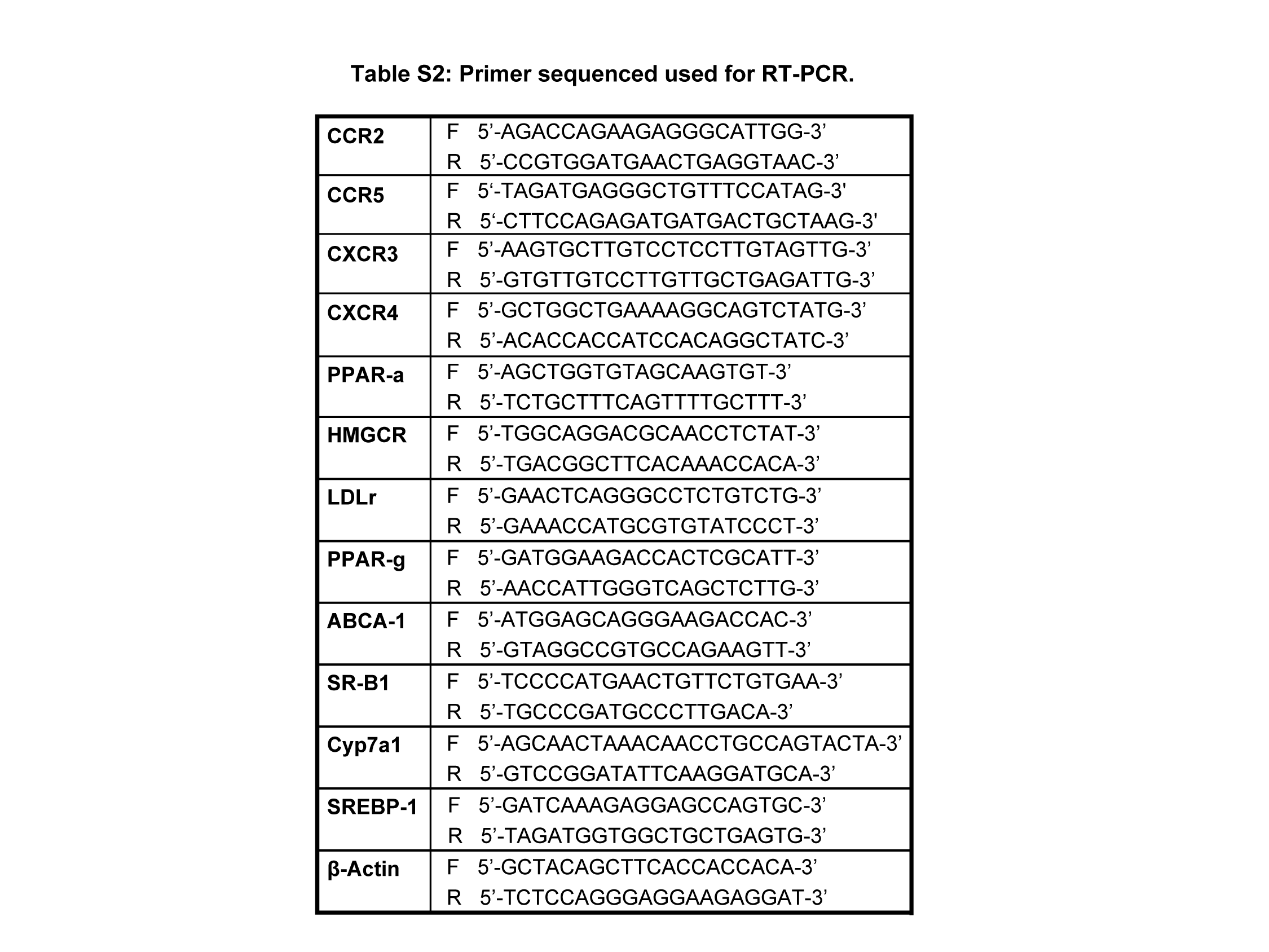

Supplement: Table S2 — Primer sequenced used for RT-PCR. (TIF) [file pone.0056253.s004.tif]
