# Supplementary material for: Maternal Diet Supplemented with Methyl-Donors Protects against Atherosclerosis in F1 ApoE−/− Mice
Source: PLoS One. 2013 Feb 21;8(2):e56253. doi: 10.1371/journal.pone.0056253 (PMC3578836; doi:10.1371/journal.pone.0056253)
Supplement: Table S1 — Diet composition. (PDF) [file pone.0056253.s003.pdf]

Table S1: Diet composition[1,2]

Ingredient	Control	MS
Amino Acids, g/kg		
L-Alanine	4.02	4.02
L-Arginine HCl	13.9	13.9
L-Asparagine	6.90	6.90
L-Aspartic Acid	4.02	4.02
L-Cystine	2.50	2.50
L-Glutamic Acid	46.0	46.0
Glycine	26.8	26.8
L-Histidine HCl, monohydrate	5.17	5.17
L-Isoleucine	9.43	9.43
L-leucine	12.8	12.8
L-Lysine HCl	20.7	20.7
*L-Methionine	4.30	11.8
L-Phenylalanine	8.63	8.63
L-Proline	4.02	4.02
L-Serine	4.02	4.02
L-Threonine	9.40	9.40
L-Tryptophan	2.07	2.07
L-Tyrosine	5.75	5.75
L-Valine	9.43	9.43
Carbohydrates, g/kg		
Sucrose	350	350
Corn Starch	150	150
Maltodextrin, Lo-Dex 10	150	150
Cellulose (Solka Floc)-non-Nutritive Fiber	30.0	30.0
Vitamins, mg/kg		
Niacin (Vit B3)	45.0	45.0
Calcium pantothenate (Vit B5)	24.0	24.0
Pyridoxine HCL (Vit B6)	10.5	10.5
Thiamine HCL (Vit B1)	9.00	9.00
Riboflavin (Vit B2)	9.00	9.00
*Folic acid (Vit B9)	3.00	13.5
Biotin (Vit B7)	0.30	0.30
*Cobalamin (Vit B12) (0.1% in mannitol)	62.5	1560
A-tocopherol (Vit E) (500 IU/g)	225	225
Retinyl palmitate (Vit A) (500,000 IU/g)	12.0	12.0
Cholecalciferol (Vit D3) (500,000 IU/g)	3.00	3.00
Phylloquinone (Vit K1)	1.12	1.12

Table S1: Diet composition continue[1,2]…

Ingredient	Control	MS
Salts and minerals, g/kg		
$Ca(H_2PO_4)_2 \cdot H_2O$	8.20	8.20
$CaCO_3$	13.5	13.5
MgO	1.95	1.95
$CuCO_3$	25.7	25.7
$FeSO_4 \cdot 7H_2O$,	0.21	0.21
Na_2SeO_3 (0.044% in sucrose)	1.25	1.25
*$ZnSO_4 \cdot 7H_2O$	0	0.72
$ZnCO_3$	0.058	0.058
KH_2PO_4	8.75	8.75
Potassium Citrate	0.98	0.98
NaCl	2.59	2.5
K_2SO_4	1.63	1.63
Ferric Citrate	0.212	0.212
$MnCO_3$	0.022	0.022
KIO_3, *mg/kg*	0.00035	0.00035
Na_2SeO_4, *mg/kg*	0.00036	0.00036
$(NH_4)_6Mo_7O_{24} \cdot 4H_2O$, *mg/kg*	0.00028	0.00028
$Na_2SiO_3 \cdot 9H_2O$, *mg/kg*	0.0508	0.0508
LiCl, *mg/kg*	0.00061	0.00061
Boric Acid, *mg/kg*	0.0028	0.0028
NaF, *mg/kg*	0.0022	0.0022
NH_4VO_3, *mg/kg*	0.00023	0.00023
$2NiCO_3 \cdot 3Ni(OH)_2 \cdot 4H_2O$, *mg/kg*	0.00011	0.00011
Others, g/kg		
Soybean Oil	80.0	80.0
*Betaine, anhydrous	0	0.015
*Choline Chloride	1.35	19.4
TBHQ, antioxidant	0.02	0.02
Total energy, (% cal)		
Protein	17.6	18.8
Carbohydrate	64.0	62.1
Fat	18.4	19.1

[1]Diets are modifications of TD.99366.

[2]Diet ingredient concentrations are presented to 3 significant digits. More precisely defined concentrations are available from Harlan Teklad.

*** Denote the ingredients that were significantly different between MS and control TD diet.**
